# Host Response to SARS-CoV2 and Emerging Variants in Pre-Existing Liver and Gastrointestinal Diseases

**DOI:** 10.3389/fcimb.2021.753249

**Published:** 2021-10-25

**Authors:** Baibaswata Nayak, Geetanjali Lal, Sonu Kumar, Chandan J. Das, Anoop Saraya, Shalimar

**Affiliations:** ^1^ Department of Gastroenterology, All India Institute of Medical Sciences, New Delhi, India; ^2^ Department of Radiodiagnosis, All India Institute of Medical Sciences, New Delhi, India

**Keywords:** SARS-CoV2, COVID-19, variants of concern, emerging variants, gastrointestinal disease, liver, manifestations

## Abstract

**Background:**

Novel coronavirus SARS-CoV2 is evolving continuously with emergence of several variants of increasing transmission capabilities and pandemic potential. Generation of variants occurs through accumulation of mutations due to the RNA nature of viral genome, which is further enhanced by variable selection pressures of this ongoing pandemic. COVID-19 presentations of SARS-CoV2 are mainly pulmonary manifestations with or without mild gastrointestinal (GI) and hepatic symptoms. However, the virus has evolved beyond pulmonary manifestations to multisystem disorder due to systemic inflammation and cytokine storm. Definitive cause of acute or late onset of inflammation, infection in various organs, and host response to emerging variants lacks clarity and needs elucidation. Several studies have reported underlying diseases including diabetes, hypertension, obesity, cardio- and cerebrovascular disorders, and immunocompromised conditions as significant risk factors for severe form of COVID-19. Pre-existing liver and GI diseases are also highly predominant in the population, which can alter COVID-19 outcome due to altered immune status and host response. We aim to review the emerging variants of SARS-CoV2 and host response in patients with pre-existing liver and GI diseases.

**Methods:**

In this review, we have elucidated the emergence and characteristic features of new SARS-CoV2 variants, mechanisms of infection and host immune response, GI and hepatic manifestation with radiologic features of COVID-19, and outcomes in pre-existing liver and GI diseases.

**Key Findings:**

Emerging variants of concern (VOC) have shown increased transmissibility and virulence with severe COVID-19 presentation and mortality. There is a drastic swift of variants from the first wave to the next wave of infections with predominated major VOC including alpha (B.1.1.7, UK), beta (B.1.351, South Africa), gamma (B.1.1.28.1, Brazil), and delta (B1.1.617, India) variants. The mutations in the spike protein of VOC are implicated for increased receptor binding (N501Y, P681R) and immune escape (L452R, E484K/Q, T478K/R) to host response. Pre-existing liver and GI diseases not only have altered tissue expression and distribution of viral entry ACE2 receptor but also host protease TMPRSS2, which is required for both spike protein binding and cleavage to initiate infection. Altered immune status due to pre-existing conditions results in delayed virus clearance or prolonged viremia. Even though GI and hepatic manifestations of SARS-CoV2 are less severe, the detection of virus in patient’s stool indicates GI tropism, replication, and shedding from the GI tract. COVID-19-induced liver injury, acute hepatic decompensation, and incidences of acute-on-chronic liver failure may change the disease outcomes.

**Conclusions:**

The changes in the spike protein of emerging variants, immunomodulation by viral proteins, and altered expression of host viral entry receptor in pre-existing diseases are the key determinants of host response to SARS-CoV2 and its disease outcome.

## Introduction

Novel coronavirus with the earliest reported pneumonia-like symptoms originated in the Wuhan City of China in December 2019, hence was designated as 2019-nCoV (Phelan et al., 2020), and the resulting outbreak was termed as coronavirus disease 2019 (COVID-19) by the World Health Organization (WHO). Subsequently, the International Committee on Taxonomy of Viruses (ICTV) renamed the virus as severe acute respiratory syndrome coronavirus 2 (SARS-CoV2) after its genome sequencing and phylogenetic analysis, which revealed 79.5% genome identity with SARS-CoV. From December 2019 till July 29, 2021, WHO has reported 195,886,929 confirmed cases of COVID-19, with 4,189,148 reported deaths worldwide. Despite its apparent correlation of origin, COVID-19 is distinct from symptoms associated with other coronaviruses such as SARS-CoV, 2003, and the Middle East respiratory syndrome by (MERS-CoV). Clinical presentation of COVID-19 varies from asymptomatic to severe such as acute respiratory distress syndrome (ARDS), multisystem inflammatory syndrome (MIS), fever, cough, dyspnea, fatigue, and pneumonia (Guan et al., 2020). In addition, loss of taste and smell, myalgia, conjunctivitis, and skin rash have also been associated with atypical and early presentation of COVID-19. Severe presentation of COVID-19 requires immediate medical attention, whereas asymptomatic presentations go unnoticed, resulting in virus dissemination and spread. SARS-CoV2 infection also presents with gastrointestinal (GI) manifestations such as diarrhea, nausea, vomiting, and abdominal discomfort. Despite the low severity ascribed to GI symptoms, the detection of virus in patient’s stool is an indication of virus replication, shedding, and GI tropism.

With emerging variants of SARS-CoV2 contributing towards enhanced infectivity and transmission as well as reports of multiple organ involvement, COVID-19 has transformed from being a respiratory disease to multisystemic disorder. Since the onset of this pandemic, association of severity of COVID-19 with old age and pre-existing comorbidities in patients has been observed. Various studies have reported that underlying diseases such as diabetes mellitus, hypertension, cardio- and cerebrovascular disorders, obesity, coronary heart disease, HIV infection, cancer, and other immunocompromised conditions are significant risk factors for developing a severe form of COVID-19, which may turn fatal (Fathi et al., 2021; Ng et al., 2021). In addition to the respiratory and GI system, involvement of the hepatobiliary system either as a pre-existing condition or consequence of treatment may also exacerbate disease progression. The contribution of indirect hepatic injury owing to cytokine storm and systemic inflammation towards COVID-19 severity currently lacks clarity (Hunt et al., 2020). Current studies suggest a relatively mild disease discourse for patients presenting with GI symptoms, but the effect of pre-existing GI and liver diseases on the prognosis of COVID-19 or resulting long COVID disease remains largely inconclusive. An in-depth analysis of case studies for a longer duration is required to accurately suggest the contribution of underlying GI and liver diseases towards altered disease outcomes. We have reviewed emerging variants of SARS-CoV2 and host response in patients with pre-existing gastrointestinal and liver diseases. Pre-existing comorbidities, including hepatic and gastrointestinal diseases, may have a role in severe presentations altering the disease outcome.

## SARS-CoV2 Infection

SARS-CoV2 is an enveloped positive-stranded RNA virus that belongs to the genus *Betacoronavirus* of the family Coronaviridae. The viral genome is 29,870 nucleotides long (Ref. NC_045512), which encodes five typical open reading frames (ORF) known as ORF1ab polyprotein (7096 amino acid, aa), Spike (S) glycoprotein (ORF2, 1273-aa), Envelope (E) protein (ORF4, 75-aa), Membrane (M) protein (ORF5, 222-aa), and Nucleocapsid (N) protein (ORF9, 419-aa) and six accessory protein-coding ORFs known as ORF3a, ORF6, ORF7a-b, ORF8, and ORF10 (Wu et al., 2020; Yadav et al., 2020). The major non-structural polyprotein ORF1ab encodes viral replicase complexes, including non-structural proteins (NSP 1-16), mainly processed by viral proteases.

SARS-CoV2 infection begins with the entry of the virus through binding of spike protein to cell entry receptor ACE2 (angiotensin-converting enzyme 2) and its cleavage by transmembrane protease serine subtype 2 (TMPRSS2) (Lu et al., 2020). Viral entry is followed by uncoating of the genomic RNA, translation of the first open reading frame (ORF1ab), and its processing by viral proteases into several non-structural proteins (nsp 1-16), including viral replicase RdRp (RNA-dependent RNA polymerase). Further, viral genomic RNA replicates through viral RdRp, and synthesis of viral structural proteins (S, N, M, and E) occur, necessary for viral assembly and release for subsequent infection.

## Host Immune Response and Immunomodulation by SARS-CoV2

Host innate and adaptive immune response to the viral infection determines the disease’s clinical course and viral clearance. The innate immune system is the first line of defense against viral infections. A diverse set of immune cells such as macrophages, dendritic cells (DCs), neutrophils, natural killer (NK) cells, and innate lymphoid cells (ILCs) sense viral infection through recognition of viral PAMPs/DAMPs (Pathogen/Damage Associated Molecular Patterns) using pattern recognition receptors (PRRs). The PAMPs majorly include viral nucleic acids (ss/ds RNA), proteins, lipids, and other viral components. The PRRs are mainly Toll-like receptors (TLRs), the NOD-Like receptors (NLRs), the RIG-I-Like receptors (RLRs), the C-type lectin receptors (CLRs), and inflammasomes. This PAMP-PRR interaction activate multiple innate immune signaling pathways such as TLR/TRIF/MyD88/IRF3/7- IFN, RLR/RIG-1/MDA5/NFkB- IFN/IL, NLR/NLRP/AIM2/Inflammasome/IL for the production of interferon and cytokines/chemokines (Seth et al., 2006). Activation and priming of innate and adaptive immune responses result in pathogen clearance and recovery. But the viruses can evade host immune responses, rendering the host susceptible to infection. Evasion of host immune response is an early occurrence initiated with viral entry, translation of first ORFs, and subsequent immunomodulation by viral proteins (Chan and Gack, 2016).

**Figure 1:**
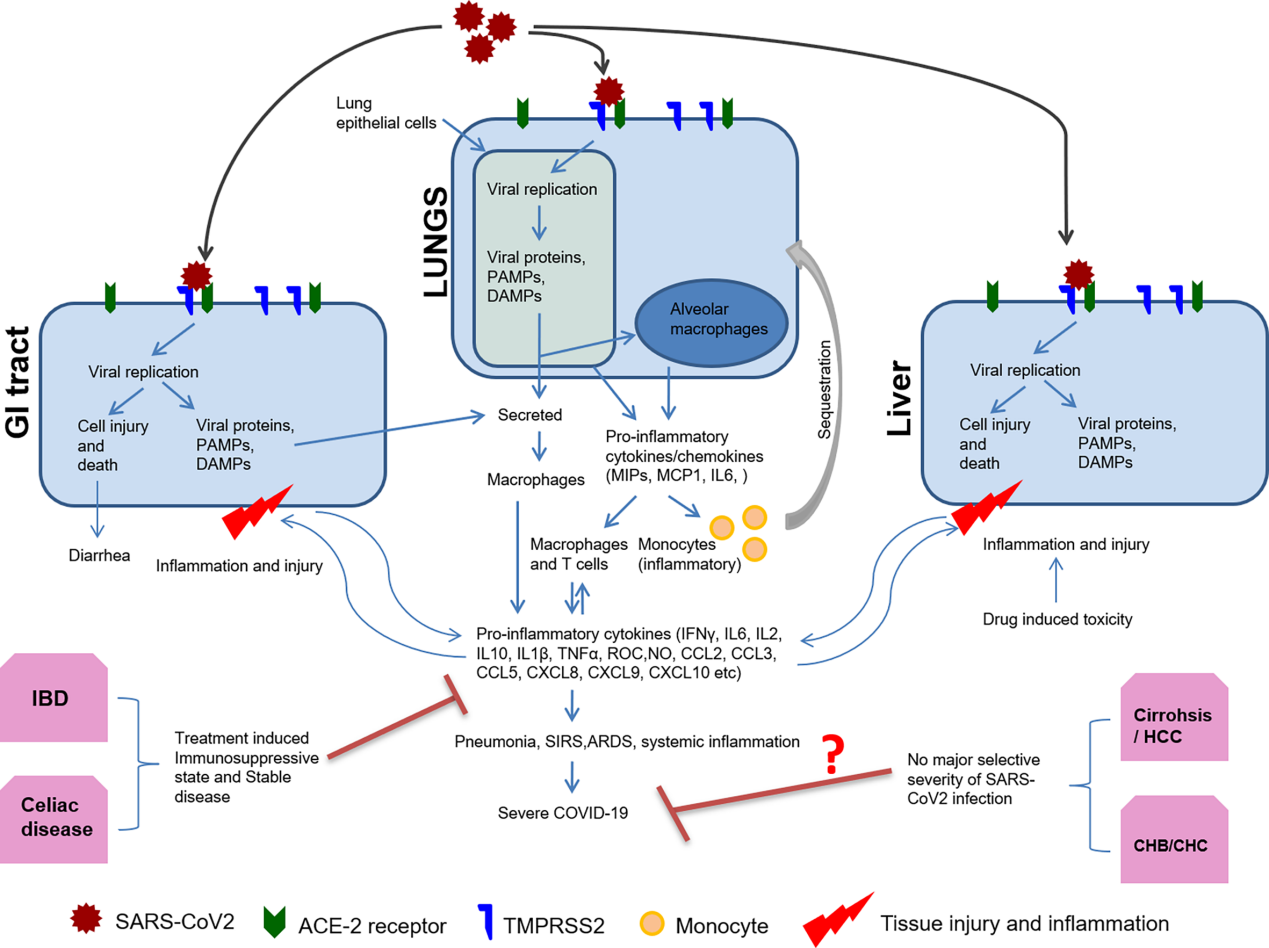
Host immune response to SARS-CoV2 and organ-specific immunomodulation. Organ specific expression of viral entry receptor ACE2 and host protease TMPRSS2 determine SARS-CoV2 tropism and replication. Host cell mounts immune response by sensing viral replication and its intermediates through PAMPS or DAMPS. Subsequently, there is antigen presentation, the release of pro-inflammatory cytokines/chemokines, and activation of the adaptive immune response. Monocytes are also sequestered to the site of inflammation by chemotaxis and differentiate into macrophages forming the pro-inflammatory milieu. This results in pneumonia, ARDS, SIRS, and systemic inflammation. Pre-existing disease conditions including liver and GI tract may affect COVID-19 outcome as summarized in this figure.

During the initial phases of infection, the virus evades the host’s innate immune system through immunomodulation by viral proteins, which leads to productive infection and unchecked viremia. SARS-CoV2 non-structural proteins such as nsp1, PLpro, 3CLpro, nsp10, and nsp16 may suppress host antiviral response, mainly type I IFN response, facilitating virus replication. Later, host may recognize viral structural proteins to mediate inflammatory response (**Figure 1**). Both T and B cell epitopes are extensively mapped to the structural proteins including S, N, M, and E protein of SARS-CoV2 (Liu et al., 2017). However, the role of all viral proteins for immunomodulation are not yet clear. For SARS-CoV2 infection, the initial immunosuppressive phase is characterized by lymphopenia, T cell exhaustion, and inadequate adaptive immune response (Diao et al., 2020). Host recognition of more viral proteins mounts severe inflammatory response at a later stage as characterized by a cytokine storm with neutrophil, monocyte/macrophage infiltration, and activation (Jacques and Apedaile, 2020). Generally, during viral infections, lymphocytes inhibit overactive innate immune responses, but lymphopenia during SARS-CoV2 infection may result in an increased release of cytokines such as interleukin (IL)-6, IL-10, IL-2, and IFN (Musa, 2020). This aggravated inflammatory response is an accentuated immune response, like SIRS, and implicated in multisystem aggravation contributing towards the severity of COVID-19 in patients (**Figure 1**).

## Gastrointestinal Manifestations of COVID-19

**Figure 2:**
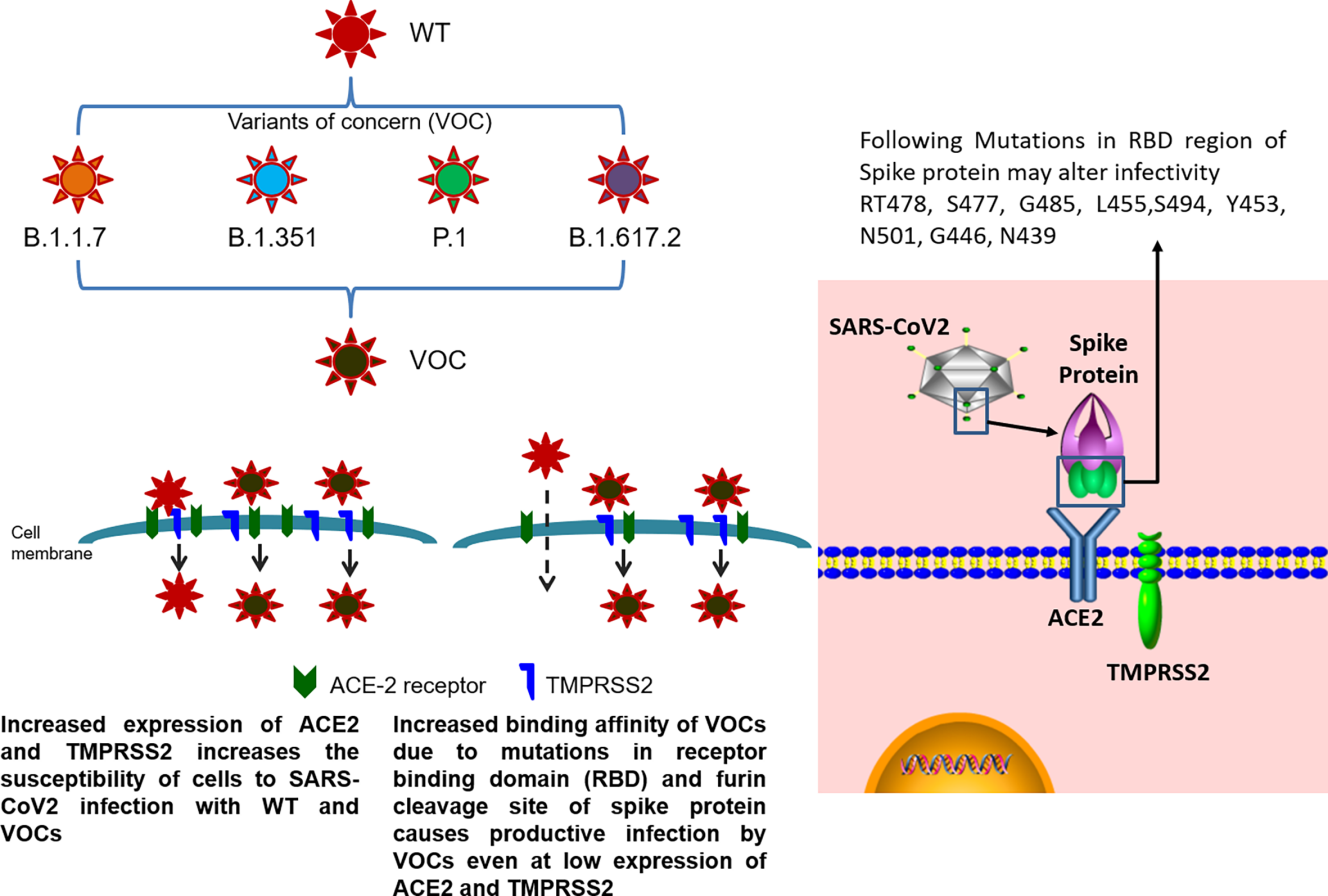
Infective potential of wild-type SARS-CoV2 and its VOC. SARS-CoV2 infection occurs by the receptor-mediated entry of the virus into the host cells using ACE2 receptor in the presence of TMPRSS2 protease. Higher expression of ACE2 receptor and TMPRSS2 on the cell membrane of host cells causes internalization of both wild-type SARS-CoV2 and its VOC. In cells with low levels of ACE2 and TMPRSS2 expression, the mutations present in the receptor binding domain (RBD) of the spike protein (RT478, S477, G485, L455, S494, Y453, N501, G446, and N439) of VOC allows for efficient binding of the variant to the host receptors even at low expression levels as compared to the wild-type virus, contributing towards the enhanced transmissibility of VOC.

COVID-19 was primarily considered a respiratory disease, but it has now evolved into a multisystem disorder with transmission *via* inhalation and oral ingestion (Roberts et al., 2020). As discussed earlier, COVID-19 disease affects various organs with no quantifiable way of predicting the affected systems with any certainty. SARS-CoV2 infection presenting itself as GI symptoms such as anosmia, diarrhea, vomiting, and abdominal pain, with or without respiratory symptoms, has widely been reported in infected patients (Smyk et al., 2020). A recent study with a large cohort of 1,099 COVID-19 patients showed that diarrhea occurred in 3.8% and vomiting in 5.0% of the cases (Wong et al., 2020). The prevalence of GI manifestations in COVID-19 patients could be as high as 50%, as approximately 50% of patients with COVID-19 have detectable virus in their stool till 27.9 days of infection (Ouali et al., 2020). The frequency of GI symptoms significantly varies depending on the geographical area, studied population, and time of assessment of the COVID-19 patients (Papa et al., 2020; Redd et al., 2020). About 17% of COVID-19 pneumonia patients experienced pancreatic injury defined by any abnormality in amylase or lipase as reported in a recent study (Patel et al., 2020). The cause of injury may be attributed to the direct cytopathic effects of SARS-CoV2 or indirect systemic inflammatory and immune-mediated cellular responses, resulting in organ damage or secondary enzyme abnormalities (Patel et al., 2020). The pancreatic injury could also occur due to circulating microthrombi causing an ischemic injury in the pancreas (Eketunde et al., 2020). GI tropism of SARS-CoV2 is attributed to the presence of ACE2 entry receptor throughout the GI tract with the greatest functional role in the small bowel and colon. Increased ACE2 expression has been observed in the enterocytes of the proximal to the distal region of the small intestine, making it more vulnerable to infection (Smyk et al., 2020). Another key player is the host protease that cleaves viral spike protein into S1 and S2 subunits that are required for attachment of the virion to both the ACE2 receptor and the cell membrane. The distribution of these proteases varies across the GI tract and probably contributes to the lack of correlation between the expression levels of ACE2 receptors and the degree of infection (**Figure 2**). Viral nucleocapsid protein has also been detected in the cytoplasm of gastric, duodenal, and rectal epithelium, indicating viral replication at these sites (Wong et al., 2020). Viral RNA also has been found in esophageal, gastric, duodenal, and rectal biopsies, but only in the patients with severe disease, suggesting that the presence of SARS-CoV2 in GI tissue is associated with a more severe disease course (Almeida and Chehter, 2020). Conversely, several studies associate presentation of GI symptoms with milder form of disease (Nobel et al., 2020; Papa et al., 2020). The current lack of clarity regarding whether GI symptoms lead to a severe or mild form of COVID-19 requires further studies and needs observation of large cohorts of patients for more assertive conclusions.

## Hepatic Manifestations of COVID-19

Hepatic manifestations of COVID-19 have been observed as a marked rise of transaminases in the blood indicative of liver injury. Several studies have reported abnormal alanine aminotransferase (ALT) and aspartate aminotransferase (AST) levels with an elevation of serum bilirubin (Guan et al., 2020). Initially, a study from Wuhan, China, reported liver manifestations in 43 out of 99 patients (Chen et al., 2020). Later, a study involving a larger (n=1,099) cohort of patients from China has shown elevated serum levels of ALT, AST, and total bilirubin in 22, 21, and 10% of the cases, respectively. Comparison of non-severe *vs* severe cases has shown higher levels (ALT 19.8 *vs* 28.1%, AST 18.2 *vs* 39.4%, and bilirubin 9.9 *vs* 13.3%) in severe COVID-19 patients (Guan et al., 2020). Hepatic dysfunction in these severe COVID-19 patients is also associated with activation of coagulative and fibrinolytic pathways, reduced platelet counts, and increase in ferritin levels, neutrophil counts, and neutrophil-to-lymphocyte ratios (Wang et al., 2020). Hepatic dysfunction either due to replication of virus in liver or by other mechanisms is lacking of histological correlation. COVID-19 patients’ post-mortem liver biopsies have shown microvesicular steatosis, hepatocyte degeneration, lobular focal necrosis, and neutrophil infiltration, but the mechanisms of liver injury in these patients remain uncertain (Lu et al., 2020). The mechanisms of hepatic injury in COVID-19 patients can be virus-induced cytopathic effects during its replication in hepatocytes, systemic inflammation-mediated immune injury through cytokine storm, pneumonia-associated hypoxia, or drug-induced hepatotoxicity (Xu et al., 2020). In addition, viral replication in liver may be more extensive as hepatic distribution of ACE-2 receptor is not limited to hepatocytes, but also expressed in the cholangiocytes and in the endothelial layer of small blood vessels of liver. Additionally, expression of ACE-2 receptor is much higher in cholangiocytes (59.7%) than hepatocytes (2.6%), which suggests that viral entry and replication in cholangiocytes may also dysregulate the liver function (Chai et al., 2020). Cholangiocytes are known to play important roles in liver regeneration, and immune response (Banales et al., 2019), and damage to these cells may cause liver injury during SARS-CoV2 infection.

## Radiologic Features of GI and Hepatobiliary System in COVID-19

**Figure 3:**
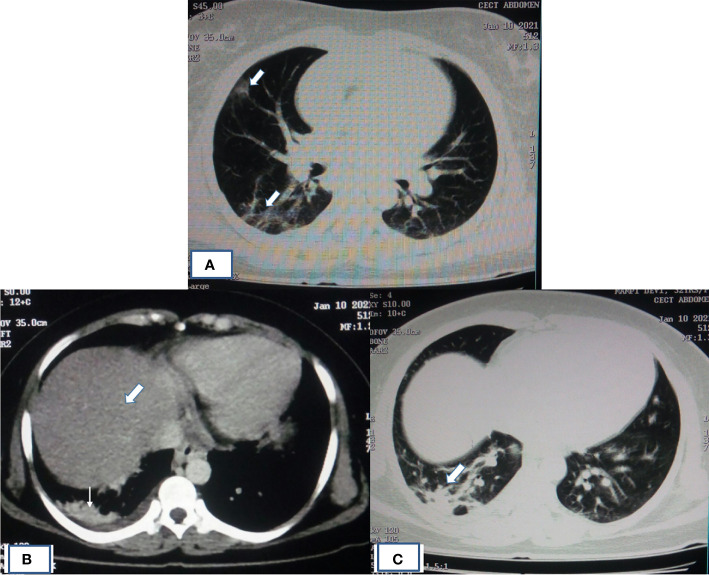
HRCT of patient showing typical imaging manifestations of COVID-19 pneumonia and fatty infiltration of liver. **(A)** HRCT of chest showing pneumonia in the form of peripheral, multifocal consolidative and ground-glass opacities as indicated by thick white arrow. **(B)** Mediastinal window image of same COVID-19 patient with altered LFT had shown fatty infiltration of liver (thick white arrow) with opacity in the right lung base (thin white arrow). **(C)** Corresponding lung window image showing ground-glass haziness of COVID-19 pneumonia (thick white arrow).

Imaging manifestations of COVID-19 in organs other than the respiratory system is attributed to the viral entry receptor expression, local organ specific virus replication, and its cytotoxic effects. Typical imaging manifestations of COVID-19 pneumonia observed in CT scan are peripheral, multifocal consolidative and ground-glass opacities as observed in high-resolution CT (HRCT) scan (**Figures 3A**, **C**). In contrast, manifestations in other organs are mainly attributed to derangement of the renin-angiotensin system due to death of cell expressing ACE2 elevating angiotensin-II and reduced angiotensin 1–7, which further potentiates endothelial inflammation, thrombosis, and hypertension (Roberts et al., 2020). Abdominal imaging in COVID-19 patients is generally performed to evaluate the cause of abdominal pain. Radiologic findings on abdominopelvic CT are small bowel thickening, solid organ infarctions, and vascular thrombosis (Goldberg-Stein et al., 2020). The proposed mechanisms of bowel wall thickening could be SARS-CoV2 binding to bowel epithelial cells, leading to mucosal inflammation and prothrombotic state (Ahmad et al., 2021). This binding can decrease ACE2 receptor expression, resulting in dysregulation of B0AT1 (broad neutral amino acid transporter, SLC6A19), which influences, COVID-19-associated diarrhea (Barbosa da Luz et al., 2020). Other potential mechanisms are altered microbiota homeostasis, enterocyte damage, release of virulent proteins or toxins, and hypercoagulable state (Suryana et al., 2021). Bowel-wall thickening can occur non-specifically and also due to neoplastic, inflammatory, infectious, or ischemic conditions (Macari and Balthazar, 2001). Other causes of abdominal pain in COVID-19 patients are presented with epiploic appendagitis due to inflammation and venous thrombosis (Bashari et al., 2020). Other related manifestations such as bowel wall hyper-enhancement, pneumatosis, portal venous gas- and fluid-filled colon have been described in literature (Bhayana et al., 2020).

**Figure 4:**
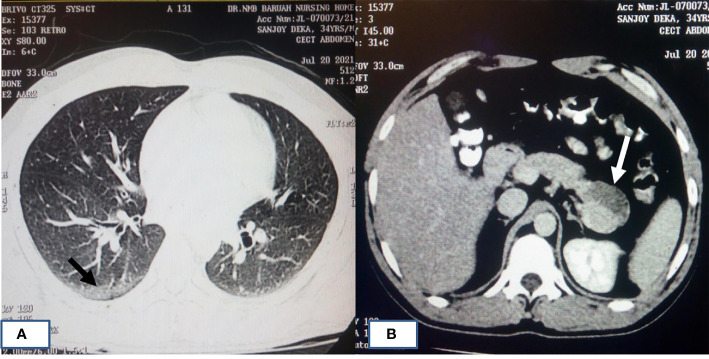
HRCT chest of patients with COVID-19 pneumonia and acute pancreatitis. **(A)** A 34-year-old male who had cough and painful abdomen showing typical imaging manifestations of COVID-19 pneumonia in the peripheral basal segment of right lower lobe (black arrow). **(B)** CECT abdomen image of same patient showing hypodensity in the tail of pancreas (white arrow) suggestive of acute necrotizing pancreatitis.

**Figure 5:**
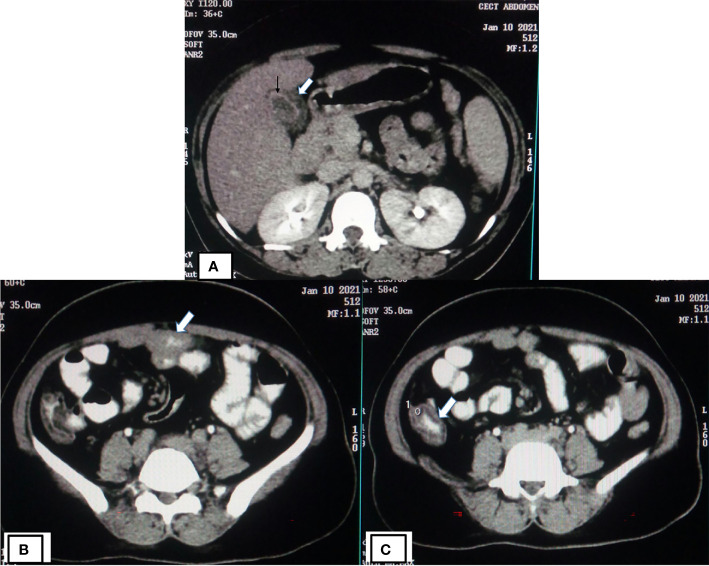
CECT of COVID-19 patients showing gall bladder, small and large bowel wall thickening. **(A)** CECT abdomen of COVID-19 patients (same patient as shown in **Figure 3**) showing gall bladder wall thickening (thick white arrow) likely due to edema and mucosal enhancement (black arrow). CECT abdomen of same patient showing small bowel (thick white arrow) wall thickening **(B)** and large bowel (thick white arrow) wall thickening **(C)**.

Almost 40% of COVID-19 patients have abnormal liver function tests (LFT) on admission, and this LFT abnormalities are associated with high fever, increased C-reactive protein, and longer hospital stay (Fan et al., 2020). Liver dysfunction mostly correlates well with imaging. Patient with altered LFT’s may show fatty infiltration in the liver as evidenced in **Figure 3B** (Fan et al., 2020). Several COVID-19 patients with abdominal pain also have shown imaging findings similar to acute pancreatitis (**Figure 4**) (Saeed et al., 2020). In an ultrasonography-based study, gallbladder wall thickening, presence of pericholecystic fluid and sludge were reported in 3, 3, and 60% of COVID-19 patients, respectively (Bhayana et al., 2020). Definite cholecystitis with gallbladder distension and wall edema was also reported in 25% COVID-19 (Goldberg-Stein et al., 2020). Gallbladder wall edema in COVID-19 patients may be a reflection of direct hepatocellular damage due to the virus or by a reactive inflammatory response such as systemic inflammatory response syndrome or Kawasaki-like multisystem inflammatory syndrome (Ahmad et al., 2021). CECT abdomen and CT angiography can show gall bladder wall thickening and edema (**Figure 5A**), small (**Figure 5B**) and large (**Figure 5C**) bowel wall thickening, vascular thrombosis and solid organ infarctions, pneumatosis, portal venous gas- and fluid-filled colon (Bhayana et al., 2020). Imaging plays a pivotal role in diagnosing various complications of GI and hepatobiliary system and may play a significant role in prognosis of patients.

## Emerging Variants of SARS-CoV2

Full-length genome sequencing and phylogenetic analysis during this pandemic period have given insight into the genetic diversity of SARS-CoV2. High rates of mutation and emerging variants are attributed to the varying selection pressure during its global spread in the current pandemic. Several variants of this pandemic virus are emerging rapidly with altered infectivity and enhanced transmission abilities. These SARS-CoV2 variants are classified into two major lineages (A, B) with several sublineages (1, 2, 3, and 5) as per the dynamic lineage or PANGO (Phylogenetic Assignment of Named Global Outbreak) lineage classification method (Rambaut et al., 2020; Rambaut et al., 2021). Lineage-A variants mostly harbor two unique mutations (8782 C>T and 28144 T>C), which are absent in Lineage-B variants. These variants of different lineages and sublineages also lead to varying manifestations after their respective infections. Association of Lineage-A variants is mostly with self‐limiting upper respiratory infections, whereas Lineage-B variants are associated with severe lower respiratory tract infections, ARDS, and extrapulmonary manifestations (Brufsky, 2020). Several synonymous and non-synonymous mutations get accumulated in the entire genome, leading to the emergence of viral variants. Recently, emerging variants of concern (VOC) were identified by comparing their common ancestor with relevant amino acid substitutions at different ORFs. The GISAID (Global Initiative on Sharing All Influenza Data) introduced another nomenclature system for major clades, developed by Sebastian Maurer-Stroh et al., based on marker mutations (alphabet for non-synonymous and number for synonymous substitutions). The hCOV-19 Metadata (2,564,149 sequence submission, as of August 1, 2021) in GISAID is now classified into 10 clades (S, L, V, G, O, GH, GR, GV, GK, and GRY) using specific combinations of genetic markers. The early splits of phylogenetic clades were S (marker C8782T, T28144C and NS8-L84S) and L (C241, C3037, A23403, C8782, G11083, G26144, T28144). Further, L clade evolved to V (G11083T, G26144T, NSP6-L37F + NS3-G251V) and G (C241T, C3037T, A23403G, and S-D614G); and later G clade evolved into GH (C241T, C3037T, A23403G, G25563T includes S-D614G + NS3-Q57H), GR (C241T, C3037T, A23403G, G28882A includes S-D614G + N-G204R), GV (C241T, C3037T, A23403G, C22227T includes S-D614G + S-A222V), and GK. Recently, GR into GRY (C241T,C3037T, 21765-21770del, 21991-21993del, A23063T, A23403G, G28882A includes S-H69del, S-V70del, S-Y144del, S-N501Y + S-D614G + N-G204R) along with GK. The sequences that do not fall under above clades were assigned O clades (GISAID, 2021). The Nextstrain clades naming is the third nomenclature system, which follows Year-Letter nomenclature to label clades that persist for at least several months and have significant geographic spread (20% frequency—globally at any time point). These clades are also defined by signature mutations and differ in at least two positions from their parent clade. The circulating clades for the year 2019 were 19A and 19B, for the year 2020 were clade 20A-20J, and till now, the 2021 clades are emerged to 21A-21H (Bedford et al., 2021). Recently, WHO using letters of the Greek alphabet to name major VOC as Alpha, Beta, Gamma, Delta, and the variants of interest (VOI) as Kappa, Epsilon, Eta, Theta, Iota, and Lambda. In the year 2020, Alpha variant (Pango Lin, B.1.1.7/GSIAID, GRY/Nextstrain, 20I clade) emerged from UK, Beta variant (Pango Lin, B.1.351/GSIAID, GH/501Y.V2/Nextstrain, 20H clade) emerged from South Africa, and Gamma variant (Pango Lin, P.1/GSIAID, GR/501Y.V3/Nextstrain, 20J clade) emerged from Brazil, whereas Delta variant (Pango Lin, B.1.617.2, AY.1/2/3/GSIAID, G/478K.V1/Nextstrain, 21A clade) recently emerged from India in the year 2021 (WHO, 2021).

The emerging variants with D614G aa substitution in the spike protein are now dominant and circulating globally. The variants with D614G substitution do not cause severe illness but alter its infectivity, competitive fitness, and transmission as evident from laboratory studies (Hou et al., 2020). Alpha variant emerged from the UK (20I/501Y.V1) during September 2020 and has shown increased transmissibility with minimal impact on the disease severity and is now reported in several countries. This variant of B.1.1.7 lineage harbors receptor-binding domain (RBD) N501Y mutation and other mutations including 69/70 deletion, spike P681H, and ORF8 stop codon (Q27stop) mutation (Leung et al., 2021).The beta variant (20H/501Y.V2) of B.1.351 lineage harbors spike N501Y, E484K, and K417N/T mutations without 69/70 deletion and is predicted to have emerged in South Africa during October 2020 with potential of global spread (Tegally et al., 2020). It is important to note that the E484K mutation, termed as “escape mutation,” which partially aids the virus to evade host immunity, is acquired either through natural infection or vaccines. These three mutations have also been observed in gamma variant (P.1/20J/501Y.V3), which is responsible for the drastic rise in COVID-19 infections and related deaths in Manaus, Brazil, during December 2020 to early 2021. This P.1 variant has 17 unique mutations, 12 of which are present in the spike protein region where the abovementioned triplet mutations cause increased binding affinity of spike protein to ACE2 receptor, probably contributing towards its enhanced infectivity and transmissibility (Faria et al., 2021). Despite these commonalities, P.1 variant is significantly less resistant to natural infection or vaccine-induced antibody response as compared to the beta variant (B.1.351), indicating viral neutralization is not restricted to only the RBD region of virus (Dejnirattisai et al., 2021). Similar to E484K, another probable escape mutation, E484Q, has been observed in the delta variant (B.1.617, G/452R.V3) currently dominant in India and detected in more than 50 other nations. Additionally, this variant has P681R and L452R mutations in the RBD region (Cherian et al., 2021; Ferreira et al., 2021), with L452R also present in B.1.427/B.1.429 variants found in California, USA, which have been associated with increased transmissibility of virus (Deng et al., 2021). As of May 2021, three sublineages have been detected for this variant (B.1.617.1/2/3) with B.1.617.2 being the most dominant, having a unique T478K mutation absent in sublineages 1 and 3, serving as a possible route of immune evasion (Di Giacomo et al., 2021). The delta variant, B.1.617.2, has been implicated in the devastating surge of infections in India from February 2021 to May 2021. In addition to the SARS-CoV2 variants discussed above, numerous other variants are currently in circulation and will emerge due to natural selection of virus resulting in accumulation of favorable mutations for the continued survival of virus (**Figure 2**). In-depth description of all these variants remains out of the scope of this review.

## Effect of SARS-CoV2 on Pre-Existing Liver and GI Diseases

COVID-19 patients with pre-existing morbidities such as diabetes, hypertension, coronary artery disease, cancer, and pre-existing liver disease are at risk of a severe disease and death (Boettler et al., 2020; Fix et al., 2020). Pre-existing liver diseases of major concern in COVID-19 are history of chronic viral hepatitis, non-alcoholic fatty liver disease (NAFLD), alcoholic liver disease (ALD), autoimmune hepatitis, hepatic compensated and decompensated cirrhosis. The impact of pre-existing liver disease due to different etiology is not completely known. The risk of SARS-CoV2 infection in advanced chronic liver disease (CLD) may be higher due to cirrhosis-induced immunodeficiency, but conversely their immunosuppressive state may provide protection from cytokine storms, modulating disease outcome of multiorgan failure in COVID-19 (Albillos et al., 2014). The effects of SARS-CoV2 infection on different pre-existing liver and GI diseases are mentioned below and summarized in the **Table 1**.

**Table 1:** Effects of SARS-CoV2 infection on pre-existing hepatic and gastrointestinal conditions.

	Pre-existing condition	Effects of SARS-CoV2 infection	Clinical manifestations	References
Hepatic	CHB	Delayed SARS-CoV2 clearance, liver injury, incidence of ACLF, acute cardiac injury, shock	Leukopenia, erythropenia, thrombocytopenia, moderate liver injury, inflammation, HBV reactivation	(Liu et al., 2020a; Liu et al., 2020b; Mehta et al., 2020; Rodriguez-Tajes et al., 2020; Zou et al., 2020)
	CHC	Delayed SARS-CoV2 clearance, delayed recovery, late antibody response in CHC-HIV co-infection, higher cases of mortality in cirrhotic patients of metabolic origin	No significant alteration from general COVID-19 symptoms	(Butt and Yan, 2020; Muller et al., 2020; Zhou et al., 2020; Mangia et al., 2020)
	NAFLD	Increased risk of COVID-19 severity, liver damage, increased expression of ACE2 and furin receptors	Hepatic injury and failure, hepatic encephalopathy, GI bleeding.	(Ji et al., 2020; Mahamid et al., 2020; Sachdeva et al., 2020; Meijnikman et al., 2020; Mushtaq et al., 2020; Biquard et al., 2020; Singh et al., 2021)
	Cirrhosis	Increased mortality, higher rate of acute hepatic decompensation and ACLF, progressive liver injury	Hepatic decompensation, liver injury, and liver failure	(Rosenblatt and Verna, 2020; Marjot et al., 2020; Sarin et al., 2020; Shalimar et al., 2020; Bajaj et al., 2020)
	HCC	Major risk factor, increased mortality	Exacerbation of hepatic injury and severity	(Jothimani et al., 2020; Kim et al., 2020; Chai et al., 2020)
	Liver transplant	Higher risk of severity in long-term (>10 yrs) patients on immunosuppressive therapy and advanced age (>65 yrs) and comorbidities	GI symptoms (nausea, vomiting, abdominal pain diarrhea), higher ICU admissions, invasive ventilation procedures	(Fix et al., 2020; Huang et al., 2020; Webb et al., 2020; Manzia et al., 2021; Theodore et al., 2021)
Gastrointestinal	IBD	Higher mortality risk associated with advanced age (>60 yrs), comorbidities, active colonic disease, corticosteroid therapy, combination of anti-TNF and corticosteroid therapy	No significant alteration from general COVID-19 symptoms	(Hunt et al., 2020; Bezzio et al., 2020; Fiorino et al., 2020; Singh et al., 2020; Lukin et al., 2020)
	Celiac disease	No major association as risk factor for COVID-19 or its severity	No significant alteration from general COVID-19 symptoms	(Zingone et al., 2020; Gokden et al., 2020; Lebwohl et al., 2021)

CHB, chronic hepatitis B; CHC, chronic hepatitis C; NAFLD, non-alcoholic fatty liver disease; HCC, hepatocellular carcinoma; IBD, irritable bowel disease; ACLF, acute-on-chronic liver failure; HIV, human immunodeficiency virus; TNF, tumor necrosis factor; GI, gastrointestinal.

### Chronic Hepatitis B

Chronic hepatitis B (CHB) is one of the most prevalent pre-existing liver diseases that affect the disease outcome of COVID-19. Dysregulation of host innate and adaptive immune response during CHB may hinder SARS-CoV2 clearance. The median time for SARS-CoV2 clearance in CHB group was reported to be longer (21 days) than in the non-HBV group (14 days) (Liu et al., 2020a). COVID-19 patients with chronic HBV infection are more prone to liver injury and poor prognosis with increased mortality and incidence of complications such as acute-on-chronic liver failure (ACLF), acute cardiac injury, and shock (Zou et al., 2020). Additionally, the clinical presentation of COVID‐19 patients with pre-existing CHB are leukopenia, erythropenia, and thrombocytopenia, along with moderate liver injury and inflammation; however, the HBV co-infection with SARS-CoV2 did not significantly affect the outcome of COVID‐19 (Liu et al., 2020b). Hepatitis B virus reactivation is also a major concern in COVID-19 patients with chronic HBV infection (Liu et al., 2020a). This is mostly associated with COVID-19 management due to immune suppressive corticosteroid therapy or biological therapies such as IL-6 receptor antagonists in patients with current or past HBV exposure (Rodriguez-Tajes et al., 2020). Such patients require HBV DNA load monitoring and treatment with antivirals for HBV such as Entecavir or Tenofovir to reduce viral load and hepatitis B flares (Mehta et al., 2020).

### Chronic Hepatitis C

A recent study of chronic HCV-infected veteran cohort found similar SARS‐CoV2 infection rate in patient groups of varying degrees of liver fibrosis (Butt and Yan, 2020). It was also observed that virus clearance and COVID‐19 recovery were delayed with late antibody response in chronic hepatitis C (CHC) patients co-infected with human immunodeficiency virus and following liver transplantation (Muller et al., 2020; Zhou et al., 2020). Another study also found lower-risk mortality in cirrhotic CHC and DAA treated or cured CHC patients, whereas case fatality rate was higher in cirrhotic patients of metabolic origin (Mangia et al., 2020).

### NAFLD

Non-alcoholic fatty liver disease (NAFLD) is considered a greater risk for COVID-19 progression and liver damage, which is further complicated by hepatic failure, hepatic encephalopathy, and GI bleeding as reported in the initial studies during pandemic (Ji et al., 2020). Another retrospective case-control study of 71 COVID-19 patients with or without fatty liver showed a significant severe COVID-19 presentation in NAFLD as compared to non-NAFLD subjects (36.3 *vs* 10.2%, OR 3.57, 95% CI 1.22–14.48) (Mahamid et al., 2020). Pooled data analysis of NAFLD in COVID-19 patients had shown an association with a higher risk of symptomatic, severe, and progressive COVID-19. The odds ratio of pooled cases after adjusting the obesity factor was found significant for this risk (OR=2.358, 95% CI: 1.902–2.923) (Sachdeva et al., 2020). Increased expression of SARS-CoV2 entry receptor ACE-2, furin, and protease TMPRSS2 was observed in liver tissue of NAFLD patients (Meijnikman et al., 2020). Meta-analysis of array-based liver gene expression data found a positive correlation of NAFLD with the expression of ACE-2 and furin but not with the expression of TMPRSS2 (Singh et al., 2021). Other studies debated that NAFLD could predict liver injury but not mortality or disease severity of COVID-19 presentation or progression (Mushtaq et al., 2020). Another study concluded that increased ACE-2 and TMPRSS2 gene expression in liver tissue of NAFLD patients was not necessary for increased liver uptake of SARS-CoV2 (Biquard et al., 2020). Association of NAFLD with severe presentation of COVID-19 is due to the fact that patients with NAFLD tend to be obese, be diabetic, and have metabolic syndrome, which have also been associated with severe form of COVID-19.

### Cirrhosis

Pre-existing cirrhotic patients are at increased risk of SARS-CoV2 infection due to their immune dysfunction status, frequent hospitalization, and associated comorbidities such as hypertension, diabetes, and obesity (Rosenblatt and Verna, 2020). Cirrhosis is not necessarily associated with diabetes, hypertension, and obesity, unless it is due to NASH/NAFLD etiology. Outcome of SARS-CoV2 infection was studied in a large number of cirrhotic patients (n=386) by comparing with non-cirrhotic patients (n=359) in and international registry study from UK hospital network (Marjot et al., 2020). Rate of mortality in cirrhotic patients has shown to be increased according to the severity of cirrhosis (Child-Pugh class A, 19%; B, 35%; and C, 51%). Higher rate of acute hepatic decompensation and acute-on-chronic liver failure occurred in patients with cirrhosis following SARS-CoV2 infection. Poor outcome to SARS-CoV2 infection in patients with cirrhosis was observed in the APCOLIS study (Sarin et al., 2020), as well as one study from our center (Shalimar et al., 2020). In the APCOLIS study, a higher rate of progressive liver injury (57%) and mortality (43%) was observed in decompensated cirrhotic patients following SARS-CoV2 infection. About 20% of cirrhotic patients presented with either acute-on-chronic liver failure or acute decompensation following infection. Similarly, from our center, high mortality (42.3%) was observed in cirrhotic patients superimposed with COVID-19 than historical cirrhotic control (23.1%) (Shalimar et al., 2020). Another multicenter study observed similar mortality risk in patients compared to superimposed cirrhosis and COVID-19, cirrhosis alone, or COVID-19 alone (Bajaj et al., 2020).

### Hepatocellular Carcinoma

Most patients with hepatocellular carcinoma (HCC) have underlying chronic liver disease, and therefore, they fall under this high-risk category and are likely to have worse outcome (Jothimani et al., 2020). A multicenter study from USA had observed HCC as an independent risk factor for higher overall mortality with a hazard ratio (HR) of 3.31 (95% CI,1.53–7.16) (Kim et al., 2020). SARS-CoV2 entry receptor ACE-2 gene expression in cancer and its impact on overall survival (OS) and disease-free survival (DFS) were recently studied (Chai et al., 2020). Higher ACE-2 expression predicted better outcome of HCC with disease-free survival, indicating a dual role of ACE-2 function predicting outcome.

## Liver Transplant and the Risk of COVID-19 Severity

SARS-CoV2 pandemic has brought with it new considerations in the cases of transplant procedures. In case of transplant patients, their immunocompromised condition may have a two-pronged effect of SARS-CoV2 infection, such as increased viremia and decreased chances of systemic inflammation. Increased risk of developing a severe pulmonary disease in liver transplant (LT) patients following SARS-CoV2 infection was not observed compared to general COVID-19 patients (Bhoori et al., 2020; D’Antiga, 2020). However, the risk of severity and mortality increased up to 23% in LT patients who were on long-term (>10 years) maintenance immunosuppressive therapy (Fix et al., 2020; Huang et al., 2020) and those with comorbidities such as >65 years age, diabetes, hypertension, and obesity (Webb et al., 2020). LT success in COVID-19 patients is high when both donor and recipients have neutralizing SARS-CoV2 antibodies prior to LT (Manzia et al., 2021). It was also observed that a large proportion of LT patients with COVID-19 were presented with GI symptoms such as nausea, vomiting, abdominal pain, and diarrhea compared to non-LT COVID-19 patients. These patients also required higher rate of ICU admissions (28 *vs* 8%) and invasive ventilation procedures (20 *vs* 5%) (Theodore et al., 2021). It should be noted that the level and nature of immunosuppression in LT patients may hinder the development of anti-SARS-CoV2 immunity (Theodore et al., 2021). Current knowledge regarding the safety of LT procedures on COVID-19 patients and the expected outcome of patients infected with SARS-CoV2 post-LT remains ambiguous. To draw clear conclusions, long-time follow-up studies are required in large cohorts of LT patients.

## GI Diseases

### Irritable Bowel Disease

IBD, being the chronic inflammation of GI tract, presents an acute challenge during the time of COVID-19 pandemic. The widespread use of immune-modulators and biological agents for complete remission and mucosal healing in Crohn’s disease and ulcerative colitis may contribute towards the progression of SARS-CoV2 infections. Various international organizations involved in research and care for IBD patients have reached the consensus of continuation of immune-active therapies with strict disinfection and social distancing measures to ablate the risk of relapse in future. International registry SECURE-IBD, to monitor outcomes of IBD patients worldwide, has reported a low fatality rate for the 6,262 registered patients from 33 countries. It did implicate age, comorbidities, steroid and mesalamine therapy with ICU admissions and death. Highest death rate of 12–25% was observed in patients >60 years of age. Mortality also increased in patients having more than one comorbidities (15–33%) and taking oral/parenteral steroids (13%), Budesonide (7%), mesalamine/sulfasalazine (6%), azathioprine and methotrexate monotherapy (6%). Conversely, anti-TNF or anti-interleukin 12/23 monotherapy only had 1–2% risk of adverse outcomes. This risk significantly increased in case of combination therapy of anti-TNF with other corticosteroids (Hunt et al., 2020). Various other studies with regards to IBD patients have reported on the prevalence of COVID-19, risk assessment, severity of disease, and mortality (Bezzio et al., 2020; Fiorino et al., 2020); and a general consensus suggests no significant rise in severity or mortality due to IBD. But advanced age, comorbidities, active colonic disease (Singh et al., 2020), and use of corticosteroid therapy (Lukin et al., 2020) are contributing risk factors. Additionally, the effect of IBD medications on the expression of ACE-2 and TMPRSS2 in ileum and colon and their contribution towards increased susceptibility to local or systemic disease are being explored (Bangma et al., 2020). Studies have shown increased ACE-2 and TMPRSS2 expression in active colon disease and ileal inflammation, respectively (Nowak et al., 2020). Currently, no association has been established with increased ACE-2 expression and risk of SARS-CoV2 infection in IBD patients.

### Celiac Disease and COVID-19

Celiac disease (CeD) is an autoimmune multiorgan disease affecting the small bowel of patients in a genetically predisposed manner. It is characterized by hyper-responsiveness of innate and adaptive immune system to gluten. Studies have established increased susceptibility of CeD patients to invasive pneumococcal disease (Simons et al., 2017) and viral infections such as influenza (Marild et al., 2010) and varicella zoster (Ludvigsson et al., 2017). In the current scenario, studies involving patient-response-based data collection and analysis have been done to determine the risk associated with CeD towards susceptibility and severity of COVID-19. A large CeD subset (n= 40,963) from nationwide histology-based population of patients with GI diseases in Sweden, ESPRESSO, was identified, and no significant increase in COVID-19-based hospitalizations, severity, or mortality was observed as compared to the control group (n= 183,892) (Lebwohl et al., 2021). Another study from Italy, involving 171 CeD patients on gluten-free diet, also suggests no major association of CeD with increased risk of SARS-CoV2 infection or severity (Zingone et al., 2020). Similarly, a questionnaire-based study conducted with 101 CeD patients in Turkey also suggests no association of CeD with COVID-19 susceptibility in patients on gluten-free diet as compared to non-CeD population (Gokden et al., 2020). Current data suggest no association of CeD with COVID-19 risk, but majority of these studies are limited in their scope due to the design of studies being unable to identify asymptomatic SARS-CoV2 infections and no long-term analysis of associated debilitations.

## Emerging Variants and Disease Outcome in Patients With Pre-Existing Liver-GI Diseases

The effects of emerging variants on the clinical outcome can be studied by comparing outcomes of COVID-19 patients in the first wave *versus* the second wave of SARS-CoV2 infections in India. Second wave surge was defined after March, 15, 2021, where emerging delta variant was widespread in India (Kar et al., 2021). Recently, our study compared the outcome of patients with clinical presentations of cirrhosis and COVID-19 patients admitted during the first (Shalimar et al., 2020) and second wave of the pandemic in India. It was observed that the clinical presentation with severe COVID-19 infection was more frequent in the second wave than the first wave of infections (51.5 *vs.* 25.0%; p=0.006). However, both the subgroups had similar duration of hospital stay and mortality.

## Host Gene Expressions in Liver and GI Tissue of SARS-CoV2-Infected Patients

Several studies have shown transcriptome analysis using tissue samples of COVID-19 patient or cell line infected with SARS-CoV2 for understanding host immune response and expression of viral entry receptor. Single-cell RNA sequencing analysis (accession no: GSE164547) of cells in human respiratory epithelium had shown variable expression of host entry molecules (ACE2, TMPRSS2, and FURIN) in different cell types, including ciliated, goblet, club, alveolar type 1 (AT1), and AT2 cells. Abundant expression of ACE2, TMPRSS2, and FURIN was observed on the apical side of ciliated cells, which support massive replication of SARS-CoV2 during early stage of infection (Ahn et al., 2021). Transcriptomic analysis (GSE148697) of SARS-CoV2-infected lung organoids enriched with permissive AT2 cells has shown upregulation of chemokines and cytokines (CXCL2, CCL2, CXCL3, IL1A, BCRC3, AADAC, and ATPB4) without upregulation of type I/III interferon signaling (Han et al., 2020). However, differential expression study in postmortem lung tissue of COVID-19 patients *vs* healthy controls (GSE147507) had shown increased expression of Type I IFN genes (IFNA4, IFNA6, IFNA10) and significant enrichment of the common Type I and Type II Interferon stimulating genes including IFNA2, IFNB1, and IFNG genes. Increased expression of antiviral innate immune response genes (IFIH1, DDX58, EIF2AK2, OAS2) and decreased expression of its negative regulators (IRF2BP1, SKIV2L) were also observed (Daamen et al., 2021). Transcriptome (GSE161881) analysis of SARS-CoV2-infected Vero E6 cells at 8 h post-infection has shown upregulations of antiviral genes (IFIT1, ZC3HAV1), NF-κB targets (CCL5, CXCL10), and ER stress response (DDIT3, PPP1R15A, GADD45B) genes (Zhang et al., 2021).

Few transcriptomic studies in extrapulmonary organ have been reported for SARS-CoV2 infection. Differential expression of genes (DEGs) in liver tissues of severe COVID-19 (GSE150316) and non-Covid 19 (GSE112356) patients have shown differential upregulation of host genes implicated in tissue remodeling (DNAJB1/hsp40, IGF2, EGFR, and HDGF), liver inflammation, and fibrosis such as metalloproteinases (MMP-3, 16,17, TIMP-1, 2, 4), collagens (COL6A3, 18A1-AS1, 20A1, 24A1, EC12, 13A1, 22A1, COLGALT2), and VCAM1. These genes are also upregulated in liver fibrosis, cirrhosis, and NAFLD. Downregulated genes such ACAD11, CIDEB, GNMT, GPAM, and cytochrome P450 (CYP450) family members are mostly implicated in metabolic pathways, mitochondrial function, and xenobiotic/drug metabolism (Hammoudeh et al., 2021). Expression profile observed in COVID-19 samples (GSE147507) has similar DEGs as seen with other diseases and comorbidities such as liver cirrhosis, influenza, and colonic neoplasms. These DEGs are also positively regulated in respiratory diseases, arthritis, psoriasis, glioblastoma, Crohn’s disease, ulcerative colitis, colorectal cancer, skin diseases, pneumonia, keratosis, lupus erythematosus, esophageal cancer (Nain et al., 2021). Single-cell transcriptome analyses of colon mucosae of normal, colorectal adenoma (CRA), and colorectal cancer (CRC) patients have shown increased expression of SARS-CoV2 entry-related genes such as ACE2, CD147 (Basigin), TMPRSS2, Cathepsin B (CATB), Cathepsin L (CATL), and Furin. Expression of these entry receptor genes is found positively correlated with genes related to inflammatory cytokine-mediated signaling pathways and virus infection. Expression of these genes may be attributed to the tissue tropism of SARS-CoV2 on intestinal cells (Chen et al., 2020).

## COVID-19 Vaccine and Dilemma of Vaccination Willingness, Emerging Variants, and Pre-Existing Diseases

SARS-CoV2 vaccines received fast-track emergency use authorization due to catastrophic consequences of ongoing COVID-19 pandemic and emergence of variants. These vaccines include mRNA vaccine expressing full-length spike protein (BNT162b2, Pfizer-BioNTech and mRNA-1273, Moderna), adenoviral vectored vaccine (ChAdOx1 nCoV-19, AstraZeneca; Covishield, Serum Institute of India; JNJ-78436735 or Ad26COVS1, Johnson & Johnson; and Sputnik V or Gam-COVID-Vac, Gamaleya National Research Centre, Russia), and whole inactivated virion (Covaxin or BBV152, Bharat Biotech; BBIBP-CorV, Sinopharm; and CoronaVac, Sinovac Biotech). The vaccine safety and efficacy data till date suggest complete safety and varying levels of efficacy, which may be further enhanced by additional booster dose or combination of different vaccines. There is also hesitancy in the general population to receive vaccine. Several surveys have reported variations in the global vaccine hesitancy average rate, which was 21% in April 2020, 36% in July 2020, and later declined to 16% in October 2020. These variations are mostly attributed to socio-demographic determinants including age, gender, ethnicity, education, risk perception of infection, and vaccine safety and efficacy (Guidry et al., 2020; Joshi et al., 2021). A recent survey (VACUNEII project) on SARS-CoV2 vaccine acceptance among gastroenterologists (n=144) and inflammatory bowel disease (IBD, n=1302) patients reported willingness of 43% of patients to receive the vaccine and 43% are not sure, whereas 95% of the physicians recommended vaccine for IBD patients (Ferreiro-Iglesias et al., 2021). Another survey on celiac disease patients (n=103) has shown 30% vaccine hesitancy including refusal due to fear of adverse events or distrust on the fast vaccine production (Costantino et al., 2021). Adverse effects have been reported in very few cases including vaccination-induced autoimmune hepatitis (AIH) (Bril et al., 2021; Rocco et al., 2021) and hepatotoxicity or drug-induced liver Injury in a patient with known history of IBD (Mann and Sekhon, 2021). Cytokine release syndrome as an adverse effect was observed after vaccination with BNT162b2 in a patient with colorectal cancer who is on longstanding anti-PD-1 monotherapy (Au et al., 2021).

The paradigm of COVID-19 vaccination in context to pre-existing liver and GI diseases does not fall under the linearity of vaccine efficacy due to the immunosuppressive state of patients either caused by the disease (cirrhosis, HCC) or the treatment (liver transplant, IBD). A recent prospective study analyzed for immune response at 4 weeks after the 2nd dose of mRNA vaccines or after the single dose of Johnson & Johnson vaccine in LT recipients (n=62) and in chronic liver disease (CLD) patients with (n=79) and without (n=92) cirrhosis. Of these, 61.3% of LT recipients and 24% of CLD patients had shown poor (undetectable <0.4 U/ml or suboptimal <250 U/ml) antibody response (Thuluvath et al., 2021). A multicenter study from China in NAFLD patients (n=381) had shown detectable antibody responses in 95.5% of patients at 14 days post-vaccination of the second dose of the alum-adjuvanted inactivated COVID-19 vaccine (Wang et al., 2021). Current vaccines indicated for protection against SARS-CoV2 have shown reduced efficacy to emerging variants such as delta as compared to the earliest strain (alpha) of SARS-CoV2 (Lopez Bernal et al., 2021).

## Drug, Target, and Future Prospective of Treatment for Severe COVID-19 With Gastrointestinal Manifestations

As discussed earlier, mild to moderate GI symptoms of nausea, vomiting, diarrhea, anorexia, and abdominal pain have been observed in COVID-19 patients. The certainty of association of these symptoms with severe progression of COVID-19 remains to be established, but COVID-19 cases presented with gastrointestinal symptoms are more likely to be severe and complicated by acute respiratory distress (ARDS), liver damage, and poor prognosis. Two separate studies (n= 141 and n=1,314) have shown that almost 74 to 86% critically ill COVID-19 patients, owing to the vigorous treatment and prolonged hospitalization, are more susceptible to develop severe gastrointestinal complications (Sun et al., 2020; Kaafarani et al., 2020). These include acute cholecystitis, acute pancreatitis, ileus and feeding intolerance, acute colonic pseudo-obstruction, hemorrhagic and ischemic colitis, and mesenteric ischemia. The cause of these severe GI manifestations may be attributed to systemic inflammation affecting extrapulmonary organ systems, side effects of drugs/antibiotics, or dysbiosis of gut microbiome or dysregulation affecting gut-liver and gut-lung axis due to SARS-CoV2 infection (Ye et al., 2020).

As first line of treatment, several repurposed drugs were explored by *in silico* studies for treatment of moderate to severe COVID-19. These drugs are broad-spectrum antivirals (remdesivir, lopinavir, ritonavir, arbidol, baloxavir marboxil, favipiravir), antiparasitic drugs (chloroquine, hydroxychloroquine, and ivermectin), immunomodulators (dexamethasone, tocilizumab, and interferon), antibiotics (azithromycin and doxycycline), and antitumor therapeutic (2-Deoxy-d-glucose, 2-DG) (Andersen et al., 2020; Mesri and Lampidis, 2021). Besides antivirals, immunomodulators are aimed to block the cytokine storm in severe COVID-19, and neutralizing antibody cocktails have been employed to prevent subsequent virus entry (Rai et al., 2021). Out of the abovementioned drugs, remdesivir and dexamethasone have shown promising clinical benefits in randomized controlled trials. The drug remdesivir is an inhibitor of RNA polymerase essential for replication of SARS-CoV2 (Pan et al., 2020). The double-blind, randomized, placebo-controlled trial of intravenous remdesivir in severe COVID-19 patients (n=1,062, remdesivir, n=541 and placebo, n= 521) found remdesivir superior to placebo in shortening the time to recovery (Beigel et al., 2020). In a real-life study of severe COVID-19 patients (n=52) receiving non-invasive ventilation, global clinical improvement was observed in 43% of patients at 12 days, 71% at 20 days, and 53% patients had de-escalation of oxygen support post-treatment with remdesivir (Simioli et al., 2021). In a controlled open-label trial of dexamethasone (n= 2,104 patients *vs* usual care n= 4,321), reduction of mortality by 3.5% was observed in patients on oxygen and reduction of mortality by 11.7% in those requiring mechanical ventilation (Horby et al., 2020).

Currently there is no effective targeted therapy for SARS-CoV2 infection. Novel drug candidates should be designed to overcome this lacuna by targeting viral proteins as well as their interacting host proteins to arrest viral infection and to promote its clearance. For this, computer-aided drug design (CADD) is an effective way of identifying lead drug molecules. The CADD uses crystallographic structure of target protein/receptor for docking of lead molecule/ligand with absolute precision and specificity. This will not only help in drug development but also help in repurposing of the available drugs for new targets. Virtual screening of small inhibitory molecules through *in silico* docking studies and its evaluation by *in vitro* assays is an effective way for quick drug discovery during virus breakthrough or pandemic. Molecular docking and molecular dynamic (MD) simulation studies have been performed by using HCV and HIV protease inhibitors as ligands on M3CLpro (protease) of SARS-CoV2 virus (Manandhar et al., 2021). Major SARS-CoV2 target proteins are the spike or S-protein; two proteases, main protease (Mpro) and papain-like protease (PLpro) enzymes, helicase and RNA-dependent RNA polymerase (RdRp) (Cavasotto and Di Filippo, 2020). Host viral entry receptor ACE2 and host protease TMPRSS2 also have been used as targets (Wu et al., 2020). Availability of crystallographic structure of these target proteins may lead to discovery of new drugs with high specificity.

## Conclusion

The SARS-CoV2 infection has taken the world by storm. The pandemic is yet to reach a plateau, with the incidences rising and newer populations getting infected. However, physicians, researchers, and scientists have left no stone unturned to understand the pathogenesis of this multisystem-afflicting disease, find effective therapies, and contain the pandemic. While the disease primarily affects the respiratory system, the liver injury does pose problems in the management of COVID-19 patients. Both direct virus-mediated cytopathic effects and indirect immune-mediated, drug-induced, or hypoxic states are probably responsible for causing and perpetuating the liver injury. However, a word of caution: Transaminitis in patients with COVID-19 should not be overly investigated. Only in patients with suspicion of a cholestatic pattern of injury, investigations like ultrasonography and magnetic resonance cholangiopancreatography may be performed. Besides, although it may sound slightly premature, in view of the recent case report, clinicians should also, in this era of COVID-19 infection, keep in mind that acute non-icteric hepatitis may be the virus’s initial presentation prior to the development of respiratory symptoms (Wander et al., 2020). As of now, no major association of risk has been ascribed to either GI symptoms or pre-existing GI diseases towards COVID-19 susceptibility. But detection of SARS-CoV2 genome in fecal matter of COVID-19 patients with GI symptoms should be treated with caution as a probable feco-oral transmission route. Recent literature suggests a major role of age and comorbidities in severe outcome of COVID-19 as seen in case of IBD patients. Role of immune-modulatory medications used for treatment of pre-existing conditions also plays a decisive role in determining the trajectory of COVID-19 progression. It should be evaluated on an individual basis until a common consensus supported by exhaustive data has been reached. As new evidence trickles in and more facts come to light, we will be in a better position to understand and tackle liver injury in COVID-19. Intensive monitoring and individually tailored approach are the need of the hour to treat patients with severe liver injury or patients with pre-existing liver diseases.

## Author Contributions

BN: Review concept, draft writing. GL: Draft writing, image preparation. SK: Literature review, draft writing. CD: Radiological feature writing and data. AS: Proof correction, draft writing. S: Proof correction, draft writing. All authors contributed to the article and approved the submitted version.

## Funding

AIIMS intramural funding support (A-COVID-36) for COVID-19 research.

## Conflict of Interest

The authors declare that the research was conducted in the absence of any commercial or financial relationships that could be construed as a potential conflict of interest.

## Publisher’s Note

All claims expressed in this article are solely those of the authors and do not necessarily represent those of their affiliated organizations, or those of the publisher, the editors and the reviewers. Any product that may be evaluated in this article, or claim that may be made by its manufacturer, is not guaranteed or endorsed by the publisher.
